# Associations between early emotional and behavioral problems and subsequent mental disorders in children – Findings from a Swedish cohort study

**DOI:** 10.1371/journal.pone.0338866

**Published:** 2026-01-07

**Authors:** Johanna Dahlén, Anton Dahlberg, Helena Fabian, Natalie Durbeej

**Affiliations:** Child Health and Parenting (CHAP), Department of Public Health and Caring Sciences, Uppsala University, Uppsala, Sweden; FSBSI Scientific Research Institute of Neurosciences and Medicine: FGBNU Naucno-issledovatel'skij institut nejronauk i mediciny, RUSSIAN FEDERATION

## Abstract

**Purpose:**

To explore the associations between early emotional and behavioral problems, measured by the Strengths and Difficulties Questionnaire (SDQ), and subsequent mental disorders among children in a Swedish context.

**Methods:**

This cohort study used data on emotional and behavioral problems in children aged 3–5 years old (n = 6,646) from the Focus study in Uppsala Region, Sweden. The data included emotional and behavioral problems using the SDQ rated by mothers (n = 6,616), fathers (n = 6,385) and preschool teachers (n = 5,079). The cohort was followed from 2013 to 2022, with psychiatric diagnoses collected from the National Patient Register. Associations between emotional and behavioral problems and later mental disorders were explored using unadjusted and adjusted Cox regression models.

**Results:**

The proportion of children identified with emotional and behavioral problems, based on the SDQ total difficulties score, was 9.4% according to mothers, 12.2% according to fathers and 7.6% according to teachers. Altogether, 9.2% (n = 611) of the children were diagnosed with a mental disorder during the follow-up period. In unadjusted models, the risk of a mental disorder was significantly higher among children with emotional and behavioral problems, as rated by mothers (HR: 3.59), fathers (HR: 2.67) and teachers (HR: 2.95). This elevated risk remained significant in adjusted models for ratings by mothers (AHR: 3.66), fathers (AHR: 2.66) and teachers (AHR: 3.06). Children with difficulties in the hyperactivity and inattention subscale of the SDQ showed a significantly increased risk of being diagnosed with attention-deficit hyperactivity or conduct disorder when rated by mothers (AHR: 4.19), fathers (AHR: 3.21) and teachers (AHR: 4.88).

**Conclusion:**

Early emotional and behavioral problems identified using the SDQ are associated with subsequent mental disorders in children in Sweden. These results reinforce the importance of early assessment of children’s emotional and behavioral functioning to enable early identification and intervention for children at risk.

## Introduction

Mental health problems in children have become a global public health concern, impacting millions of young individuals. These issues contribute significantly to the overall disease burden, accounting for one-fifth of all disease-related disabilities among children and young adults aged 5–24 years [1]. Emotional and behavioral problems are the most common mental health concerns [2], affecting nearly one in six preschool-aged children [3].

Emotional problems are characterized by internalizing symptoms, such as excessive worry, fear, and sadness, which affect a child’s emotional well-being. In contrast, behavioral problems are externalizing in nature, including symptoms such as hyperactivity, impulsivity, defiance, or aggressive behavior [2]. Such problems have been shown to remain stable during childhood, often persisting into adulthood and negatively impacting a child’s social and emotional development [4–7]. Emotional and behavioral problems can also develop into diagnosable mental disorders, such as anxiety, depression, and attention-deficit hyperactivity disorder (ADHD) [8–10]. Mental disorders affect nearly 14% of adolescents worldwide, leading to significant suffering and disability [1]. This progression from emotional and behavioral problems to mental disorders often results in increased healthcare utilization, highlighting the long-term impact on both individuals and healthcare systems [11–13]. Moreover, caring for a child with a mental disorder places a significant emotional, financial, and psychological burden on families, often leading to increased stress, social isolation, and reduced quality of life for caregivers [14]. Therefore, early identification and interventions are crucial for improving long-term outcomes for affected children [5,13,15].

Emotional and behavioral problems are more common in younger children compared to older children [10]. Behavioral problems are more prevalent among boys, while emotional problems, such as excessive worry, are more common among girls [16]. Previous research show that socioeconomic disadvantages, such as low parental education, single-parent households, and having immigrant parents, are associated with a higher risk of emotional and behavioral problems and mental disorders in children [16–20]. Moreover, studies indicate that younger parental age increases the risk of externalizing problems and a formal diagnosis of attention-deficit hyperactivity disorder in children [21,22].

In Sweden, the Child Health Services (CHS) are responsible for monitoring the physical and mental health and development of children [23]. With almost complete national coverage, the CHS reaches 99% of children under 6 years of age for regular health check-ups, based on national guidelines [24]. During these visits, children are assessed by a specialist nurse and, at key developmental stages, also seen by a general practitioner in joint evaluations. The aim of the CHS is to identify problems in children’s physical, mental and social health, and to facilitate interventions such as additional CHS visits or referral to specialist care [25]. Although there is an increasing demand for evidence-based methods within the Swedish CHS, no national standard currently exists for evaluating emotional and behavioral problems in children [19,25].

Although previous international research suggests a link between emotional and behavioral problems and subsequent mental disorders in children, no study has yet explored this association within a Swedish context. Additionally, there is a notable gap in the international research regarding the association between emotional and behavioral problems in early childhood, i.e., specifically during the preschool years, and mental health problems later in life. Exploring this association could enhance early identification and intervention efforts, thereby potentially reducing the long-term consequences of mental health problems in children. Therefore, the aim of this study was to examine the association between early emotional and behavioral problems and subsequent mental disorders among children in Sweden. Based on previous research [8–10], we hypothesized that children identified with emotional and/or behavioral problems would have an increased risk of developing subsequent mental disorders.

## Methods

### Study design

This study used a longitudinal cohort study design based on data from the *Children and Parents in Focus study* (the Focus study), which aimed to investigate young children’s mental health in a Swedish context [26]. The Focus study included a cohort of children aged 3–5 years in the Uppsala Region, Sweden, with data collected between August 1, 2013 and October 31, 2017 [27]. In the current study, the cohort was followed from when the SDQ-ratings were completed to December 31, 2022 using psychiatric diagnosis data obtained from the National Patient Register (NPR).

### Participants and procedure

Children in the Focus study were recruited in connection to their annual developmental assessments and health check-up at their local Child Healthcare Center. Prior to these appointments at ages 3, 4, and 5, families received an invitation letter as part of routine practice. Parents of all participating children gave their written informed consent on behalf of their children prior to inclusion in the study. The consent included authorization for both parents and preschool teachers to provide information on the children. The invitation letter included study information, consent forms, and study questionnaires. If the parents agreed to participate in the study, they were asked to complete one form each, sign the consent form, and bring the completed materials to the annual health visit. In addition to the questionnaires completed by both parents, a separate set for the child’s preschool teacher was included and distributed by the parents. The questionnaires included items on child demographics, parental socioeconomic background, and the Strengths and Difficulties Questionnaire (SDQ), which was to assess children’s emotional and behavioral problems [28]. The letter and accompanying forms and questionnaires were available in Swedish, as well as in the three most commonly spoken languages among the immigrant population in the Uppsala Region: English, Somali, and Arabic [26].

**Fig 1:**
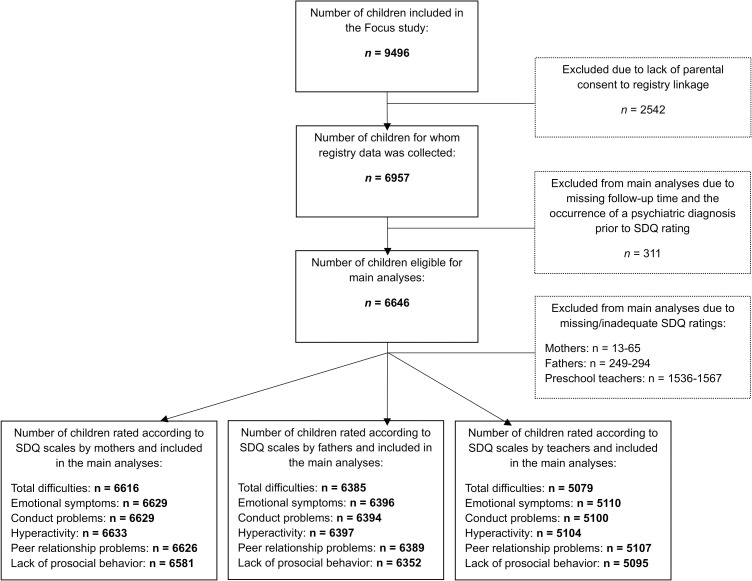
Participant flowchart.

The Focus study data included 9,496 individual children, and register data were collected for 6,957 of them. Since data collection spanned over a period of 4 years (2013–2017), some children were represented at several time points. For the purposes of this study, we used only the earliest rating to focus on emotional and behavioral problems in the youngest children. Of the 6,957 children, those who had been diagnosed with a mental disorder prior to the SDQ assessment or who had missing follow-up data were excluded (n = 311). This resulted in a final sample of 6,646 children eligible for the main analyses (Fig 1).

### Data sources

#### Emotional and behavioral problems.

SDQ ratings provided by mothers, fathers, and preschool teachers were used to identify children’s emotional and behavioral problems. These ratings, drawn from the parent and teacher versions of the SDQ, served as exposures in this study. The SDQ is a 25-item instrument comprising five subscales: emotional symptoms, conduct problems, hyperactivity/inattention, and peer relationship problems. The sum of four of these subscales provides a total difficulties score. The fifth subscale, prosocial behavior, is a positive scale and is not included in the total difficulties score. The items are scored on a 3-point Likert scale, ranging from 0 to 2 (0 = not true, 1 = somewhat true, and 2 = certainly true), with a maximum total score of 40 points [28]. To define emotional and behavioral problems, we used both continuous SDQ scores and cut-off values based on the 90th percentile according to Swedish norms for preschool children [29]. For exposures, we included both the total difficulties score and the individual subscale scores. The SDQ has demonstrated acceptable psychometric properties in young children [30–32], including Swedish norms demonstrating acceptable validity and reliability [29].

#### Psychiatric diagnoses.

This study used psychiatric diagnoses based on the 10th revision of the International Classification of Diseases (ICD-10 codes) as outcomes. For the collection of ICD-codes, the personal identity numbers of the children in the cohort were linked to the Swedish NPR, which was established in 1964 and has had national coverage of diagnoses in both specialized outpatient and inpatient settings since 1987. The NPR’s long standing national coverage and high validity make it well suited for population-based research [33]. Both primary and secondary diagnoses were included to define children’s mental disorders. The outcomes examined were: any mental disorder (ICD-10 codes: F00-F99), any anxiety disorder (ICD-10 codes: F40-F48), or any attention-deficit hyperactivity or conduct disorder (ICD-10 codes: F90-F91) during the follow-up period.

#### Confounders.

Data on confounders were obtained from self-reported information in the Focus study and included child age (3, 4, or 5 years) and sex (girl or boy), parental age (younger than 35 years or 35 and older), parental country of birth (Sweden or another country), marital status (married/cohabiting or single/living apart/other), and educational level (higher or lower level). These confounders were included based on previous research demonstrating their associations with mental health problems in children and adolescents [10,16–20].

### Statistical analyses

Means, standard deviations, ranges, frequencies, and proportions were used for descriptive purposes. Chi-square tests were conducted to compare children with and without any mental disorder in terms of identified emotional and behavioral problems and confounding variables.

Kaplan-Meier curves were evaluated graphically to assess the proportional hazards assumption. No violations were found, indicating the data were suitable for survival analysis. The associations between identified emotional and behavioral problems and children’s mental disorders were explored using both unadjusted and adjusted Cox regression models, with days as the underlying time scale. Follow-up time was calculated from the date each SDQ was completed, by each rater, to the date of diagnosis as recorded in the NPR, or until December 31, 2022, for children without a diagnosis. The mean follow-up time was approximately 7.2 years, corresponding to 2,631 days for mothers’ ratings, 2,626 for fathers’ ratings, and 2,645 days for teachers’ ratings. Exposures were defined as scoring above or below the clinical cut-off on the SDQ total difficulties scale and its subscales [29]. Given the reversed scoring on the prosocial behavior subscale in the analyses, we refer to it as “lack of prosocial behavior”. Separate analyses were conducted based on ratings from mothers, fathers and teachers. Children lacking SDQ ratings from for example one of the parents and the teacher were then only included in the analysis with the other parent’s SDQ rating as exposure.

The outcomes of this study included three different sets of mental disorders: 1) any mental disorder, 2) any anxiety disorder, and 3) any attention-deficit hyperactivity or conduct disorder. Separate Cox regression models were run for each of the three outcomes using continuous SDQ scales scores, with both the total difficulties score and the subscales as exposures. Crude and adjusted hazard ratios (HRs and AHRs) were reported for each regression model, along with 95% confidence intervals (CIs). In the adjusted models, we included child’s age and sex, parental age, parental country of birth, marital status, and educational level as confounders. For data preparation, we used the statistical software IBM SPSS version 28, and R, version 4.2.3 was used for the analyses.

### Ethical considerations

Written consent from parents or legal guardians to track children from the Focus study using register data on ICD-codes collected from the NPR was obtained prior to this study. The study was approved by the Regional Ethical Review Board in Uppsala and The Ethics Review Authority of Sweden (document numbers 2012/437 and 2022-06316-02).

## Results

### Participant characteristics

At baseline, approximately half of the participating children were 3 years old (50.8%) (Table 1), and the gender distribution was even, with 49.5% girls and 50.5% boys. Most parents of the included children were born in Sweden, 84.1% of mothers and 81.8% of the fathers. Among mothers, 72.6% had a higher level of education, compared to 58.4% of fathers. Over 90% of both mothers and fathers were married or cohabiting. Additionally, 58.6% of mothers and 69.1% of fathers were aged 35 years or older. The proportion of children identified with emotional and behavioral problems, based on the SDQ total difficulties score, was 9.4%, when rated by mothers, 12.2% when rated by fathers, and 7.6% when rated by teachers. Of the 6,646 children in the study sample, 9.2% (n = 611) were diagnosed with a mental disorder during the follow-up. Among these 611 children, 65.8% (n = 402) were boys and 34.2% (n = 209) were girls.

**Table 1:** Description of child demographics, parental socio-demographics, children’s emotional and behavioral problems^1^, and psychiatric diagnoses^2^.

	All study participants (n = 6646)	No psychiatric diagnosis (n = 6035)	Any psychiatric diagnosis during follow-up (n = 611)	P-value^3^
**Variables**	n (%)			
**Child gender**				
Girl	3287 (49.5)	3078 (51.0)	209 (34.2)	<0.001
Boy	3359 (50.5)	2957 (49.0)	402 (65.8)	
**Child age**				
3 years	3379 (50.8)	3112 (51.6)	267 (43.7)	<0.001
4 years	1791 (26.9)	1612 (26.7)	179 (29.3)	
5 years	1476 (22.2)	1311 (21.7)	165 (27.0)	
**Maternal country of birth**				
Sweden	5591 (84.1)	5060 (83.8)	531 (86.9)	0.054
Outside Sweden	966 (14.5)	893 (14.8)	73 (11.9)	
Missing	89 (1.3)	82 (1.4)	7 (1.1)	
**Paternal country of birth**				
Sweden	5436 (81.8)	4920 (81.5)	516 (84.5)	0.040
Outside Sweden	885 (13.3)	820 (13.6)	65 (10.6)	
Missing	325 (4.9)	295 (4.9)	30 (4.9)	
**Maternal education level**				
Higher educational level^4^	4828 (72.6)	4440 (73.6)	388 (63.5)	<0.001
Lower educational level^5^	1661 (25.0)	1457 (24.1)	204 (33.4)	
Missing	157 (2.4)	138 (2.3)	19 (3.1)	
**Paternal education level**				
Higher educational level^4^	3880 (58.4)	3578 (59.3)	302 (49.4)	<0.001
Lower educational level^5^	2334 (35.1)	2063 (34.2)	271 (44.4)	
Missing	432 (6.5)	394 (6.5)	38 (6.2)	
**Maternal marital status**				
Married or cohabiting	6279 (94.5)	5718 (94.7)	561 (91.8)	<0.001
Single/living apart/other	260 (3.9)	217 (3.6)	43 (7.0)	
Missing	107 (1.6)	100 (1.7)	7 (1.1)	
**Paternal marital status**				
Married or cohabiting	6077 (91.4)	5538 (91.8)	539 (88.2)	<0.001
Single/living apart/other	227 (3.4)	186 (3.1)	41 (6.7)	
Missing	342 (5.1)	311 (5.2)	31 (5.1)	
**Maternal age** ^**6**^				
< 35 years	2663 (40.1)	2405 (39.9)	258 (42.2)	0.242
≥ 35 years	3892 (58.6)	3548 (58.8)	344 (56.3)	
Missing	91 (1.4)	82 (1.4)	9 (1.5)	
**Paternal age** ^**7**^				
< 35 years	1728 (26.0)	1558 (25.8)	170 (27.8)	0.265
≥ 35 years	4591 (69.1)	4181 (69.3)	410 (67.1)	
Missing	327 (4.9)	296 (4.9)	31 (5.1)	
**Emotional symptoms rated by mothers**				
No identified problems	5763 (86.7)	5276 (87.4)	487 (79.7)	<0.001
Identified problems	866 (13.0)	743 (12.3)	123 (20.1)	
Missing	17 (0.3)	16 (0.3)	1 (0.2)	
**Conduct problems rated by mothers**				
No identified problems	5605 (84.3)	5177 (85.8)	428 (70.0)	<0.001
Identified problems	1024 (15.4)	843 (14.0)	181 (29.6)	
Missing	17 (0.3)	15 (0.2)	2 (0.3)	
**Hyperactivity/inattention rated by mothers**				
No identified problems	5709 (85.9)	5280 (87.5)	429 (70.2)	<0.001
Identified problems	924 (13.9)	742 (12.3)	182 (29.8)	
Missing	13 (0.2)	13 (0.2)	0 (0)	
**Peer relationship problems rated by mothers**				
No identified problems	5591 (84.1)	5128 (85.0)	463 (75.8)	<0.001
Identified problems	1035 (15.6)	889 (14.7)	146 (23.9)	
Missing	20 (0.3)	18 (0.3)	2 (0.3)	
**Lack of prosocial behavior rated by mothers**				
No identified problems	6242 (93.9)	5697 (94.4)	545 (89.2)	<0.001
Identified problems	339 (5.1)	280 (4.6)	59 (9.7)	
Missing	65 (1.0)	58 (1.0)	7 (1.1)	
**Total difficulties rated by mothers**				
No identified problems	5989 (90.1)	5533 (91.7)	456 (74.6)	<0.001
Identified problems	627 (9.4)	476 (7.9)	151 (24.7)	
Missing	30 (0.5)	26 (0.4)	4 (0.7)	
**Emotional symptoms rated by fathers**				
No identified problems	5525 (83.1)	5049 (83.7)	476 (77.9)	<0.001
Identified problems	874 (13.2)	757 (12.5)	117 (19.1)	
Missing	247 (3.7)	229 (3.8)	18 (2.9)	
**Conduct problems rated by fathers**				
No identified problems	5190 (78.1)	4778 (79.2)	412 (67.4)	<0.001
Identified problems	1207 (18.2)	1027 (17.0)	180 (29.5)	
Missing	249 (3.7)	230 (3.8)	19 (3.1)	
**Hyperactivity/inattention rated by fathers**				
No identified problems	5329 (80.2)	4931 (81.7)	398 (65.1)	<0.001
Identified problems	1071 (16.1)	876 (14.5)	195 (31.9)	
Missing	246 (3.7)	228 (3.8)	18 (2.9)	
**Peer relationship problems rated by fathers**				
No identified problems	5133 (77.2)	4704 (77.9)	429 (70.2)	<0.001
Identified problems	1259 (18.9)	1099 (18.2)	160 (26.2)	
Missing	254 (3.8)	232 (3.8)	22 (3.6)	
**Lack of prosocial behavior rated by fathers**				
No identified problems	5557 (83.6)	5045 (83.6)	512 (83.8)	0.692
Identified problems	798 (12.0)	721 (11.9)	77 (12.6)	
Missing	291 (4.4)	269 (4.5)	22 (3.6)	
**Total difficulties rated by fathers**				
No identified problems	5580 (84.0)	5144 (85.2)	436 (71.4)	<0.001
Identified problems	808 (12.2)	656 (10.9)	152 (24.9)	
Missing	258 (3.9)	235 (3.9)	23 (3.8)	
**Emotional symptoms rated by teachers**				
No identified problems	4463 (67.2)	4102 (68.0)	361 (59.1)	<0.001
Identified problems	650 (9.8)	566 (9.4)	84 (13.7)	
Missing	1533 (23.1)	1367 (22.7)	166 (27.2)	
**Conduct problems rated by teachers**				
No identified problems	4427 (66.6)	4090 (67.8)	337 (55.2)	<0.001
Identified problems	676 (10.2)	569 (9.4)	107 (17.5)	
Missing	1543 (23.2)	1376 (22.8)	167 (27.3)	
**Hyperactivity/inattention rated by teachers**				
No identified problems	4491 (67.6)	4166 (69.0)	325 (53.2)	<0.001
Identified problems	616 (9.3)	497 (8.2)	119 (19.5)	
Missing	1539 (23.2)	1372 (22.7)	167 (27.3)	
**Peer relationship problems rated by teachers**				
No identified problems	4487 (67.5)	4137 (68.6)	350 (57.3)	<0.001
Identified problems	623 (9.4)	527 (8.7)	96 (15.7)	
Missing	1536 (23.1)	1371 (22.7)	165 (27.0)	
**Lack of prosocial behavior rated by teachers**				
No identified problems	4723 (71.1)	4335 (71.8)	388 (63.5)	<0.001
Identified problems	375 (5.6)	321 (5.3)	54 (8.8)	
Missing	1548 (23.3)	1379 (22.9)	169 (27.7)	
**Total difficulties rated by teachers**				
No identified problems	4576 (68.9)	4235 (70.2)	341 (55.8)	<0.001
Identified problems	506 (7.6)	404 (6.7)	102 (16.7)	
Missing	1564 (23.5)	1396 (23.1)	168 (27.5)	
**Psychiatric diagnosis**				
No psychiatric diagnosis	6035 (90.8)	6035 (100)	0 (0)	<0.001
Any psychiatric diagnosis during mothers’ follow-up	611 (9.2)	0 (0)	611 (100)	

^1^Based on Swedish SDQ norms available for preschool children.

^2^Any psychiatric diagnosis (ICD F00-F99) during mothers’ follow-up period.

^3^P-values from corresponding chi-square tests. A total of 311 ratings excluded due to missing follow-up data or due to the child receiving a psychiatric diagnosis prior to the SDQ rating.

^4^University/college degree.

^5^Not completed primary school/primary school/gymnasium or training school.

^6^M (SD, range) = 35.6 (4.6, 19–58).

^7^M (SD, range) = 38.1 (5.8, 19–72).

### Associations between children’s emotional and behavioral problems and mental disorders

Unadjusted Cox regression models showed an increased risk of being diagnosed with a mental disorder among children identified with emotional and behavioral problems, based on the SDQ total difficulties score, as rated by mothers (HR: 3.59, 95% CI: 2.99–4.32), fathers (HR: 2.67, 95% CI: 2.22–3.21), and teachers (HR: 2.59, 95% CI: 2.36–3.68). These associations remained significant in the adjusted Cox regression models: mothers (AHR: 3.66, 95% CI: 3.03–4.42), fathers (AHR: 2.66, 95% CI: 2.20–3.21), and teachers (AHR 3.06, 95% CI: 2.44–3.83) (Table 2). Further analyses of the SDQ subscales revealed that children scoring above the cut-off on any individual subscale were also at an increased risk of receiving a mental disorder diagnosis.

**Table 2:** Emotional and behavioral problems according to the SDQ^1^ and the association with any psychiatric diagnosis (ICD F00-F99).

SDQ scale	SDQ problems by informant	Unadjusted models		Adjusted models^2^	
		Events/N	HR^3^ (95% Cl)	Events/N	HR (95% Cl)
**Emotional symptoms**	**Maternal report** ^**4**^				
	No identified problems	487/5763	1.00	471/5581	1.00
	Identified problems	123/866	**1.75 (1.43–2.13)**	118/832	**1.70 (1.38–2.08)**
	**Paternal report** ^**5**^				
	No identified problems	475/5522	1.00	458/5314	1.00
	Identified problems	117/874	**1.63 (1.33–2.00)**	111/832	**1.56 (1.27–1.92)**
	**Teacher report** ^**6**^				
	No identified problems	361/4461	1.00	347/4332	1.00
	Identified problems	84/649	**1.63 (1.28–2.06)**	83/636	**1.62 (1.28–2.06)**
**Conduct problems**	**Maternal report** ^**4**^				
	No identified problems	428/5605	1.00	413/5418	1.00
	Identified problems	181/1024	**2.48 (2.09–2.95)**	176/994	**2.63 (2.20–3.14)**
	**Paternal report** ^**5**^				
	No identified problems	411/5188	1.00	394/4988	1.00
	Identified problems	180/1206	**1.97 (1.65–2.34)**	174/1156	**2.14 (1.79–2.56)**
	**Teacher report** ^**6**^				
	No identified problems	337/4425	1.00	327/4301	1.00
	Identified problems	107/675	**2.14 (1.72–2.66)**	102/657	**2.30 (1.84–2.89)**
**Hyperactivity/** **inattention**	**Maternal report** ^**4**^				
	No identified problems	429/5709	1.00	415/5531	1.00
	Identified problems	182/924	**2.94 (2.47–3.49)**	175/884	**2.74 (2.29–3.28)**
	**Paternal report** ^**5**^				
	No identified problems	397/5326	1.00	384/5118	1.00
	Identified problems	195/1071	**2.64 (2.23–3.14)**	185/1029	**2.43 (2.04–2.90)**
	**Teacher report** ^**6**^				
	No identified problems	325/4489	1.00	313/4367	1.00
	Identified problems	119/615	**2.85 (2.31–3.52)**	116/595	**2.86 (2.31–3.54)**
**Peer relationship problems**	**Maternal report** ^**4**^				
	No identified problems	463/5591	1.00	448/5418	1.00
	Identified problems	146/1035	**1.80 (1.49–2.16)**	140/991	**1.91 (1.58–2.31)**
	**Paternal report** ^**5**^				
	No identified problems	429/5133	1.00	411/4930	1.00
	Identified problems	159/1256	**1.59 (1.32–1.90)**	155/1210	**1.76 (1.46–2.12)**
	**Teacher report** ^**6**^				
	No identified problems	350/4486	1.00	336/4358	1.00
	Identified problems	96/621	**2.05 (1.64–2.57)**	95/607	**2.15 (1.71–2.70)**
**Lack of prosocial** **behavior**	**Maternal report** ^**4**^				
	No identified problems	545/6242	1.00	526/6030	1.00
	Identified problems	59/339	**2.12 (1.62–2.77)**	57/335	**2.23 (1.70–2.93)**
	**Paternal report** ^**5**^				
	No identified problems	511/5555	1.00	491/5329	1.00
	Identified problems	77/797	1.02 (0.80–1.30)	74/777	**1.32 (1.02–1.71)**
	**Teacher report** ^**6**^				
	No identified problems	388/4721	1.00	373/4590	1.00
	Identified problems	54/374	**1.79 (1.35–2.39)**	54/363	**1.83 (1.38–2.44)**
**Total difficulties**	**Maternal report** ^**4**^				
	No identified problems	456/5989	1.00	442/5803	1.00
	Identified problems	151/627	**3.59 (2.99-4.32)**	145/599	**3.66 (3.03–4.42)**
	**Paternal report** ^**5**^				
	No identified problems	435/5578	1.00	419/5363	1.00
	Identified problems	152/807	**2.67 (2.22–3.21)**	146/773	**2.66 (2.20–3.21)**
	**Teacher report** ^**6**^				
	No identified problems	341/4574	1.00	329/4444	1.00
	Identified problems	102/505	**2.95 (2.36–3.68)**	99/493	**3.06 (2.44–3.83)**

HR = hazards ratio, CI = confidence interval, Numbers in bold indicate significant associations.

^1^Based on Swedish Strengths and Difficulties Questionnaire norms available for preschool children.

^2^Analyses involving mothers’ and teachers’ responses were adjusted for child gender, child age, maternal country of birth, maternal education level, maternal marital status, and maternal age. Analyses involving fathers’ responses were adjusted for child gender, child age, paternal country of birth, paternal education level, paternal marital status, and paternal age.

^3^Participants with no identified problems were used as the reference group in all analyses.

^4^Analyses performed with mothers’ follow-up time.

^5^Analyses performed with fathers’ follow-up time.

^6^Analyses performed with preschool teachers’ follow-up time.

The total SDQ difficulties score for emotional and behavioral problems was also significantly associated with later diagnoses of any anxiety disorder. This association was significant in both unadjusted and adjusted models for ratings provided by mothers (HR: 2.62, 95% CI: 1.68–4.07; AHR: 2.33, 95% CI: 1.46–3.73) and fathers (HR: 2.01, 95% CI: 1.29–3.13; AHR: 1.82, 95% CI: 1.15–2.89). However, no significant associations were found between teachers’ ratings and diagnoses of anxiety disorders in the adjusted models (Table 3).

**Table 3:** Emotional and behavioral problems according to the SDQ^1^ and the association with any anxiety disorder diagnosis (ICD F40-F48).

SDQ scale	SDQ problems by informant	Unadjusted models		Adjusted models^2^	
		Events/N	HR^3^ (95% Cl)	Events/N	HR (95% Cl)
**Emotional symptoms**	**Maternal report** ^**4**^				
	No identified problems	88/5763	1.00	85/5581	1.00
	Identified problems	31/866	**2.38 (1.58–3.59)**	28/832	**2.1 (1.37–3.23)**
	**Paternal report** ^**5**^				
	No identified problems	99/5522	1.00	95/5314	1.00
	Identified problems	18/874	1.17 (0.71–1.93)	16/832	1.04 (0.61–1.76)
	**Teacher report** ^**6**^				
	No identified problems	67/4461	1.00	63/4332	1.00
	Identified problems	16/649	1.59 (0.92–2.74)	16/636	1.70 (0.98–2.94)
**Conduct problems**	**Maternal report** ^**4**^				
	No identified problems	90/5605	1.00	85/5418	1.00
	Identified problems	29/1024	**1.77 (1.16–2.69)**	**28/994**	**1.82 (1.18–2.79)**
	**Paternal report** ^**5**^				
	No identified problems	82/5188	1.00	78/4988	1.00
	Identified problems	35/1206	**1.84 (1.24–2.73)**	33/1156	**1.87 (1.24–2.81)**
	**Teacher report** ^**6**^				
	No identified problems	65/4425	1.00	62/4301	1.00
	Identified problems	18/675	**1.73 (1.02–2.91)**	17/657	1.62 (0.94–2.78)
**Hyperactivity/** **inattention**	**Maternal report** ^**4**^				
	No identified problems	94/5709	1.00	91/5531	1.00
	Identified problems	25/924	**1.74 (1.12–2.71)**	22/884	1.54 (0.96–2.47)
	**Paternal report** ^**5**^				
	No identified problems	86/5326	1.00	83/5118	1.00
	Identified problems	31/1071	**1.84 (1.22–2.77)**	28/1029	**1.63 (1.05–2.50)**
	**Teacher report** ^**6**^				
	No identified problems	70/4489	1.00	67/4367	1.00
	Identified problems	13/615	1.28 (0.71–2.32)	12/595	1.25 (0.68–2.32)
**Peer relationship problems**	**Maternal report** ^**4**^				
	No identified problems	92/5591	1.00	87/5418	1.00
	Identified problems	26/1035	**1.56 (1.01–2.41)**	25/991	**1.57 (1.00–2.46)**
	**Paternal report** ^**5**^				
	No identified problems	80/5133	1.00	76/4930	1.00
	Identified problems	35/1256	**1.86 (1.25–2.77)**	34/1210	**1.93 (1.28–2.89)**
	**Teacher report** ^**6**^				
	No identified problems	69/4486	1.00	66/4358	1.00
	Identified problems	14/621	1.41 (0.79–2.51)	13/607	1.37 (0.76–2.49)
**Lack of prosocial** **behavior**	**Maternal report** ^**4**^				
	No identified problems	107/6242	1.00	102/6030	1.00
	Identified problems	11/339	**1.91 (1.03–3.56)**	10/335	1.77 (0.92–3.38)
	**Paternal report** ^**5**^				
	No identified problems	92/5555	1.00	87/5329	1.00
	Identified problems	25/797	**1.79 (1.15–2.78)**	24/777	**1.91 (1.17–3.13)**
	**Teacher report** ^**6**^				
	No identified problems	75/4721	1.00	71/4590	1.00
	Identified problems	8/374	1.26 (0.61–2.61)	8/363	1.35 (0.65–2.81)
**Total difficulties**	**Maternal report** ^**4**^				
	No identified problems	93/5989	1.00	90/5803	1.00
	Identified problems	25/627	**2.62 (1.68–4.07)**	22/599	**2.33 (1.46–3.73)**
	**Paternal report** ^**5**^				
	No identified problems	90/5578	1.00	87/5363	1.00
	Identified problems	25/807	**2.01 (1.29–3.13)**	23/773	**1.82 (1.15–2.89)**
	**Teacher report** ^**6**^				
	No identified problems	72/4574	1.00	69/4444	1.00
	Identified problems	11/505	1.34 (0.71–2.53)	10/493	1.29 (0.66–2.51)

HR = hazards ratio, CI = confidence interval, Numbers in bold indicate significant associations.

^1^Based on Swedish Strengths and Difficulties Questionnaire norms available for preschool children.

^2^Analyses involving mothers’ and teachers’ responses were adjusted for child gender, child age, maternal country of birth, maternal education level, maternal marital status, and maternal age. Analyses involving fathers’ responses were adjusted for child gender, child age, paternal country of birth, paternal education level, paternal marital status, and paternal age.

^3^Participants with no identified problems were used as the reference group in all analyses.

^4^Analyses performed with mothers’ follow-up time.

^5^Analyses performed with fathers’ follow-up time.

^6^Analyses performed with preschool teachers’ follow-up time.

The risk of being diagnosed with an attention-deficit hyperactivity or conduct disorder was significantly elevated among children with identified emotional and behavioral problems, based on ratings by mothers’ (HR: 4.11, 95% CI: 3.23–5.23; AHR: 4.19, 95% CI: 3.27–5.36), fathers’ (HR: 3.26, 95% CI: 2.57–4.14; AHR: 3.21, 95% CI: 2.51–4.10), and teachers’ (HR: 4.64, 95% CI: 3.53–6.09; AHR: 4.88, 95% CI: 3.68–6.46). Scoring above the cut-offs on the individual SDQ subscales for hyperactivity and inattention or conduct problems was also associated with an increased risk of being diagnosed with an attention-deficit hyperactivity or conduct disorder (Table 4).

**Table 4:** Emotional and behavioral problems according to the SDQ^1^ and the association with any attention-deficit hyperactivity or conduct disorder diagnosis (ICD F90-F91).

SDQ scale	SDQ problems by informant	Unadjusted models		Adjusted models^2^	
		Events/N	HR^3^ (95% Cl)	Events/N	HR (95% Cl)
**Emotional symptoms**	**Maternal report** ^**4**^				
	No identified problems	270/5763	1.00	259/5581	1.00
	Identified problems	59/866	**1.48 (1.12–1.97)**	57/832	**1.46 (1.09–1.94)**
	**Paternal report** ^**5**^				
	No identified problems	261/5522	1.00	252/5314	1.00
	Identified problems	61/874	**1.52 (1.15–2.01)**	57/832	**1.42 (1.06–1.89)**
	**Teacher report** ^**6**^				
	No identified problems	193/4461	1.00	183/4332	1.00
	Identified problems	46/649	**1.64 (1.19–2.26)**	45/636	**1.60 (1.15–2.22)**
**Conduct problems**	**Maternal report** ^**4**^				
	No identified problems	210/5605	1.00	201/5418	1.00
	Identified problems	120/1024	**3.32 (2.65–4.15)**	116/994	**3.49 (2.77–4.39)**
	**Paternal report** ^**5**^				
	No identified problems	205/5188	1.00	194/4988	1.00
	Identified problems	117/1206	**2.55 (2.03–3.2)**	115/1156	**2.89 (2.29–3.65)**
	**Teacher report** ^**6**^				
	No identified problems	161/4425	1.00	154/4301	1.00
	Identified problems	77/675	**3.23 (2.46–4.23)**	73/657	**3.72 (2.8–4.94)**
**Hyperactivity/** **inattention**	**Maternal report** ^**4**^				
	No identified problems	201/5709	1.00	193/5531	1.00
	Identified problems	129/924	**4.41 (3.54–5.51)**	124/884	**3.93 (3.12–4.94)**
	**Paternal report** ^**5**^				
	No identified problems	184/5326	1.00	177/5118	1.00
	Identified problems	138/1071	**4.01 (3.21–5.00)**	132/1029	**3.62 (2.88–4.54)**
	**Teacher report** ^**6**^				
	No identified problems	148/4489	1.00	140/4367	1.00
	Identified problems	89/615	**4.63 (3.56–6.02)**	86/595	**4.64 (3.54–6.09)**
**Peer relationship problems**	**Maternal report** ^**4**^				
	No identified problems	256/5591	1.00	247/5418	1.00
	Identified problems	73/1035	**1.58 (1.22-2.06)**	69/991	**1.74 (1.33–2.28)**
	**Paternal report** ^**5**^				
	No identified problems	237/5133	1.00	225/4930	1.00
	Identified problems	83/1256	**1.49 (1.16–1.91)**	82/1210	**1.76 (1.36–2.27)**
	**Teacher report** ^**6**^				
	No identified problems	178/4486	1.00	168/4358	1.00
	Identified problems	61/621	**2.54 (1.90–3.40)**	60/607	**2.67 (1.99–3.59)**
**Lack of prosocial** **behavior**	**Maternal report** ^**4**^				
	No identified problems	298/6242	1.00	287/6030	1.00
	Identified problems	29/339	**1.83 (1.25–2.69)**	27/335	**1.94 (1.30–2.88)**
	**Paternal report** ^**5**^				
	No identified problems	283/5555	1.00	271/5329	1.00
	Identified problems	36/797	0.85 (0.60–1.20)	35/777	1.22 (0.84–1.77)
	**Teacher report** ^**6**^				
	No identified problems	201/4721	1.00	190/4590	1.00
	Identified problems	34/374	**2.14 (1.49–3.07)**	34/363	**2.15 (1.49–3.09)**
**Total difficulties**	**Maternal report** ^**4**^				
	No identified problems	236/5989	1.00	227/5803	1.00
	Identified problems	93/627	**4.11 (3.23–5.23)**	89/599	**4.19 (3.27–5.36)**
	**Paternal report** ^**5**^				
	No identified problems	223/5578	1.00	214/5363	1.00
	Identified problems	97/807	**3.26 (2.57–4.14)**	93/773	**3.21 (2.51–4.10)**
	**Teacher report** ^**6**^				
	No identified problems	161/4574	1.00	153/4444	1.00
	Identified problems	76/505	**4.64 (3.53–6.09)**	73/493	**4.88 (3.68–6.46)**

HR = hazards ratio, CI = confidence interval, Numbers in bold indicate significant associations.

^1^Based on Swedish Strengths and Difficulties Questionnaire norms available for preschool children.

^2^Analyses involving mothers’ and teachers’ responses were adjusted for child gender, child age, maternal country of birth, maternal education level, maternal marital status, and maternal age. Analyses involving fathers’ responses were adjusted for child gender, child age, paternal country of birth, paternal education level, paternal marital status, and paternal age.

^3^Participants with no identified problems were used as the reference group in all analyses.

^4^Analyses performed with mothers’ follow-up time.

^5^Analyses performed with fathers’ follow-up time.

^6^Analyses performed with preschool teachers’ follow-up time.

Analyses using continuous SDQ scale scores as exposures yielded similar results. Specifically, higher scores on both the SDQ total difficulties score and the individual SDQ subscales were associated with an increased risk of being diagnosed with a mental disorder (S1-S3 Tables). These associations remained significant in the adjusted Cox regression models, confirming the findings from the main analyses.

## Discussion

The purpose of this study was to investigate the associations between early emotional and behavioral problems and subsequent mental disorders among children in a Swedish context. Our main findings indicated that children with emotional and behavioral problems, as identified by the SDQ total difficulties score, had an increased risk of being diagnosed with a mental disorder later in life. Children who scored above the cut-off on any of the individual subscales were also at an increased risk for later mental disorders. Additionally, children with emotional and behavioral problems had an increased risk of later diagnoses of attention-deficit hyperactivity, conduct, or anxiety disorders. The highest hazard ratios in this study were observed between the SDQ hyperactivity and conduct problems subscales and subsequent attention-deficit hyperactivity and conduct disorder, respectively. These associations were significant across ratings by mothers, fathers, and teachers in both unadjusted and adjusted analyses.

Our results align with previous research that show similar associations between early emotional and behavioral problems, as measured by the SDQ, and mental disorders during adolescence. A longitudinal study conducted in Denmark, with a cohort of 2,315 children aged 5–7 years, found that the SDQ, when used with a predictive algorithm, can identify children at risk for a formal diagnosis of attention-deficit hyperactivity disorder before age 11–12 [34]. Another Danish study, using the same cohort, further examined the predictive algorithm and found moderate sensitivity for attention-deficit hyperactivity disorders but low sensitivity for emotional disorders, including anxiety disorders [35].

The analysis of continuous scores as exposures confirms our main findings and is also consistent with previous research. For instance, a longitudinal study conducted in an Australian setting demonstrated that total SDQ scores at ages 4–5 years predicted formal diagnoses of neurodevelopmental disorders, as well as self-rated mental health problems measured by the SDQ at ages 16–17 [36]. This finding is consistent with our results, indicating that higher SDQ scores are linked to an elevated risk of subsequent mental disorders.

Our results showed an elevated risk of subsequent mental disorders for children with early emotional and behavioral problems. These findings are supported by earlier research showing that mental health problems tend to persist through childhood and develop into mental disorders [4–7]. The high hazard ratios for the association between hyperactivity and conduct problems according to teachers’ SDQ ratings, and attention-deficit hyperactivity and conduct disorders, suggest that preschool teachers can provide valuable insights for identifying children at risk for such disorders. Furthermore, our results showed that emotional and behavioral problems, as rated by mothers and fathers, were associated with a subsequent anxiety disorder. However, teachers’ ratings of the total difficulties score did not show a significant association with later anxiety disorders. These discrepancies between parental and teacher ratings may be explained by natural variations in children’s behavior, depending on the context. Therefore, it is considered best practice to use multi-informants when assessing children’s emotional and behavioral problems [37,38].

Given that mental disorders remain moderately stable during preschool age [39], our findings emphasize the importance of assessing emotional and behavioral problems in children as young as 3–5 years old. Moreover, research indicates that nearly one in six preschool children in primary care settings may experience mental health problems [3]. This prevalence highlights the need to utilize primary care settings, such as the CHS, as effective arenas for evaluating and addressing mental health concerns in young children. In New Zealand and the Netherlands, the SDQ has been implemented for mental health assessment in CHS settings [40,41]. However, in Sweden, there is currently no nationally adopted method or instrument for assessing children’s emotional and behavioral problems [19]. Our findings suggest that the SDQ could be a useful instrument for identifying children at risk of developing a mental disorder later in life. Additionally, our results indicate that the hyperactivity/inattention and conduct problems subscales may be appropriate for evaluating the risk of a subsequent mental disorder, particularly attention-deficit hyperactivity or conduct disorder. This study demonstrates how emotional and behavioral problems identified as early as 3–5 years of age can be predictive of future mental disorders. It is well known that early identification and interventions are crucial for these children [11,13,15]. By integrating structured, evidence-based mental health assessments into the CHS, children could receive the support they need at an early stage, potentially mitigating negative outcomes of mental health problems. This approach could help prevent the financial and psychological strain, stress, and reduced quality of life that affect families of children with mental health problems [14].

### Strengths and limitations

The main strengths of this study include the large population-based sample and longitudinal design, where the children were followed for approximately 7 years. Another strength is the use of the SDQ to measure emotional and behavioral problems, as this instrument has demonstrated reliable psychometric properties in previous research on preschool children [29–32]. Additionally, we applied Swedish age- and gender-based cut-offs to identify these problems. SDQ ratings were collected from three different informants, providing a comprehensive assessment of the children’s emotional and behavioral problems. We also used objective data on mental disorders in the form of ICD-codes for psychiatric diagnoses, collected from Sweden’s well-established NPR. However, it is important to note that the NPR lacks data from primary care and only includes diagnoses made by physicians, excluding those made by psychologists. In Sweden, it is uncommon for children to be diagnosed with a mental disorder in primary care due to the structure of the healthcare system. This introduces a small risk of misclassification, as diagnoses made by psychologists were not captured. Furthermore, the study included children recruited in connection with their annual health check-ups within the CHS. Since the CHS has nearly complete coverage, this recruitment strategy allowed us to reach a broad range of families. The cohort was followed into adolescence, and thus not capturing mental disorders diagnosed in adulthood. Given that neurodevelopmental disorders as attention-deficit hyperactivity disorder are commonly diagnosed during adolescence, but for example mood disorders often have a later onset and are diagnosed later in life, the calculated risk of any mental disorder would possibly be higher if the children were followed into adulthood [42]. Whether the SDQ remains a useful tool among teenagers for predicting mental disorders in adulthood was not explored in our study and could warrant further investigation. Finally, by examining the association between early emotional and behavioral problems and subsequent mental disorders among children in a Swedish context, this study fills an important research gap.

Despite efforts to include families from diverse backgrounds, the study population was, to a large extent, indigenous, well-educated, and composed mainly of older parents. This selection bias means that the sample may not be fully representative of the general population in Sweden. A more diverse sample would likely have included a higher proportion of families with lower socioeconomic status, a factor that is strongly linked to mental health problems. Consequently, with a more representative cohort, we would have obtained more generalizable results and might have observed a stronger association between early emotional and behavioral problems and later mental disorders. It should also be noted that the SDQ ratings could have introduced bias due to over- or underreporting of the children’s emotional and behavioral problems.

As confounders, we included socioeconomic variables such as parental educational level and country of birth. However, we did not have data on parental income, employment status, family dynamics or conflicts. Given that such factors may be associated with mental health problems in children [17–19,43,44], their absence could be considered a limitation of the study.

## Conclusion

Early emotional and behavioral problems at 3–5 years of age are associated with subsequent mental disorders in Swedish children. Our findings suggest that the SDQ is effective in identifying children at risk for these disorders. In conclusion, this instrument could serve as a valuable screening tool for emotional and behavioral problems within the Swedish CHS, which, in turn, could facilitate early support and mental health interventions.

## Supporting information

S1 Table SDQ continuous score (total and subscales) and the association with any psychiatric diagnosis (ICD F00-F99). (XLSX)

S2 Table SDQ continuous score (total and subscales) and the association with any anxiety disorder (ICD F40-F48). (XLSX)

S3 Table SDQ continuous score (total and subscales) and the association with any attention-deficit hyperactivity or conduct disorder (ICD F90-F91). (XLSX)
